# Evaluation of the Effects of Conjunctivochalasis Excision on Tear Stability and Contrast Sensitivity

**DOI:** 10.1038/srep37570

**Published:** 2016-11-28

**Authors:** Weiqiang Qiu, Mingzhou Zhang, Ting Xu, Ziyuan Liu, Huibin Lv, Wei Wang, Xuemin Li

**Affiliations:** 1 Department of Ophthalmology, Peking University Third Hospital, Beijing, China; 2 Department of Ophthalmology, Beijing Shunyi Hospital, Beijing, China.

## Abstract

Conjunctivochalasis (CCh) disrupts tear flow and damages tear film stability. This study sought to evaluate the tear stability and contrast sensitivity of patients with CCh on whom CCh excision was performed. The study included 39 eyes from 39 patients; all patients had eyes with grade 2 or 3 CCh, underwent CCh excision, and were evaluated before and three months after the surgery. The evaluated variables included the ocular surface disease index (OSDI), the tear break-up time (TBUT), corneal fluorescein staining, corneal surface irregularity, Schirmer’s I test, the tear meniscus area (TMA), and contrast sensitivity. A follow-up of three months was achieved in 36 eyes for 36 patients. All parameters improved significantly after surgery (*p* < 0.05), except Schirmer’s I test, thus suggesting that CCh excision is an effective method for reconstructing the lower tear meniscus and improving both tear film stability and corneal surface irregularity. The results further demonstrated a simultaneous increase in contrast sensitivity after surgery.

Conjunctivochalasis (CCh) was defined by Hughes as redundant, loose, nonedematous redundant conjunctiva located between the globe and eyelid, especially at the lower fornix^1^. By disrupting the tear flow, the redundant folds of conjunctiva may induce ocular inflammation, damage the tear film stability, and aggravate dry eye symptoms^2^,^3^. Individuals with CCh may be asymptomatic or experience ocular symptoms. Previous reports have described a range of symptoms in patients with CCh, including dryness, eye pain, blurry vision, epiphora, and redness. In this patient population, over 50% of individuals experienced dryness, especially upon awakening and during reading^4^,^5^.

Despite the abundance of data investigating the association between CCh and dry eye, to our knowledge, no previous studies have simultaneously assessed changes in contrast sensitivity and the tear meniscus after surgery. To build on the current literature, we estimated the structural alteration of the lower fornix and assessed the change in the visual function before and after conjunctiva resection. It is plausible that the removal of extra conjunctiva helps to the tear reservoir reconstruction; furthermore, improves both ocular surface stability and contrast sensitivity.

## Results

This study included 39 eyes from 39 patients. The ages of the individuals ranged from 47 to 81 years. Three patients did not complete the follow-up period and were excluded from the analysis. Finally, 36 eyes from 36 patients (28 women and 8 men) were analyzed. All general patient information is delineated in Table 1. None of the patients had infections or other complications, and the surgical margin healed well in all cases.

### OSDI, corneal fluorescein staining scores, TBUT and Schirmer’s I test improved after CCh excision

All subjects with CCh mainly complained of grittiness and a foreign body sensation; however, after the surgery, various ocular surface parameters showed improvement. The OSDI significantly decreased from pre-excision to three months after the operation (58.11 ± 19.36 and 18.27 ± 11.67, *p* < 0.001) (Table 2), and statistical improvement was also observed in TBUT (1.86 ± 0.81 s and 5.53 ± 2.22 s, *p* < 0.001) and the corneal fluorescein staining scores (1.05 ± 0.54 and 0.16 ± 0.15, *p* < 0.001) (Table 2). However, Schirmer’s I test values without anesthesia did not show a significant difference before and after treatment (6.29 ± 2.93 mm and 7.92 ± 2.58 mm, *p* = 0.286) (Table 2). The corneal surface irregularity, estimated by the corneal topography, also showed improvement (0.026 ± 0.003 and 0.019 ± 0.011, *p* = 0.032) after surgery (Table 2).

### TMA increased after CCh excision

After removing the extra folds of conjunctiva, the cross-sectional TMA significantly increased from pre-operation to three months after surgery (641.28 ± 339.57 μm^2^ and 3,256.42 ± 4,884.98 μm^2^, *p* = 0.019) (Table 2). This finding suggested that removal of redundant conjunctiva increased the effective area in the meniscal tear.

### Contrast sensitivity and glare disability after CCh excision

Table 3 shows the contrast sensitivities in simulated daylight (85 cd/m^2^), night (3 cd/m^2^), and glare disability for each frequency before and after the operation. The contrast sensitivities increased significantly in all frequencies three months after the operation in simulated daylight and night conditions. In glare disability conditions, significantly improved values were found at all spatial frequencies three months after the operation.

## Discussion

According to previous studies, redundant conjunctiva result in instability of the tear film and dysfunction of the tear meniscus, which may result in disrupted tear flow, delayed tear clearance, and increased tear osmolarity^3^,^6^,^7^,^8^. All of the above factors cause ocular surface inflammation, contributing to dry eye.

Numerous surgical methods have been reported as conjunctival treatments^6^,^9^,^10^,^11^. The efficacy of surgical resection for CCh can be assessed by examining various parameters and assessing the improvement of ocular signs and symptoms. In our study, we chose to focus on established parameters, such as the OSDI, the TBUT, Schirmer’s I test, and corneal fluorescein staining. Furthermore, we also examined corneal surface irregularities to assess changes in the structure as well as the TMA and contrast sensitivity to simultaneously assess visual function.

In the current study, we observed improvements in OSDI, TBUT, and corneal fluorescein staining three months after surgical removal of the redundant conjunctiva, although no differences were observed in Schirmer’s I test, in accordance with results from previous studies^12^,^13^. Therefore, we hypothesized that the direct cause of discomfort in CCh patients might be decreased the tear film stability and that removal of extra conjunctiva might be an effective method to relieve the uncomfortable dryness and enhance the stability of the tear film. Furthermore, the results of Schirmer’s I test also suggested that aqueous deficiency has a limited causal relationship with patients’ symptoms.

The resection of redundant conjunctiva reduced the tissue occupation in the lower fornix, while simultaneously freeing up space for the tear meniscus. According to the observed outcomes, the mean value of the lower TMA increased significantly three months after surgery. These findings suggest that the excision of conjunctiva may help to restore the anatomical structure of the tear meniscus with a resulting elevation of the tear volume.

Similar to our results, results from Norihiko Yokoi *et al*. have indicated that surgery successfully reconstructed the tear meniscus at the lower lid margin in nearly 2/3 of cases; the patients’ clinical symptoms were also much improved^9^. Yokoi *et al*. have also reported that most cases showed significant improvement, although some showed no marked changes^6^.

By contrast, Gumus and colleagues have measured the cross-sectional TMA of CCh patients before and after conjunctival cauterization with anterior segment OCT and have found a slight, but not significant, decrease in the central meniscus^14^. These contrasting findings may be due to the difference of methods between Gumus’s and our study, since conjunctival cauterization changes the whole area status of conjunctiva, while the excision removes the visible relaxation of the conjunctiva. Consequently, it was observed a central TMA decrease after CCh removal. However, we included only temporal CCh, which may act as a slope, contributing to flowing of the tear towards the nasal side. In addition, the subclinical ectropion, mentioned by Gumus and colleagues, might increase the TMA in the area without CCh. Nonetheless, the sample sizes of both studies were limited, and the measurement classification also requires improvement. Thus, these differences in outcomes after the removal of CCh warrant further studies.

The current study is the first to evaluate contrast sensitivity before and after surgery. Previous studies have suggested that an unstable tear film cannot keep the precorneal surface smooth and, consequently, may have negative effects on contrast sensitivity and damage visual function^15^. According to Paulsson and Sjostrand, any irregularity in the ocular media may decrease the contrast sensitivity and cause disability glare^16^. To our knowledge, past reports on the relationship between contrast sensitivity and dry eyes, rather than CCh, have suggested that in patients with dry eyes, compared with control subjects, the contrast sensitivity is significantly reduced^17^.

We hypothesize that tear film changes in CCh may also result in ocular irregularities. Under all illuminating conditions, including glare, CCh patients showed significantly lower contrast sensitivity before surgery, although all participants’ best-corrected visual acuities were between 20/30 and 20/20 according to the Snellen eye chart. This result provides evidence of a visual decrease in CCh patients and is also in agreement with the findings of previous studies^18^. Previous studies have determined that contrast sensitivity is a more accurate evaluation of the functional visual acuity than the Snellen chart because the former measures the ability to resolve subtle spatial differences in a low-contrast situation, which, compared with the high-contrast targets in the Snellen chart, is more sensitive and similar to situations experienced in daily life^19^. By disrupting the tear flow, the redundant folds of conjunctiva may damage the tear film stability. Tear film changes in dry eye patients may cause irregularities of the corneal surfaces, and any irregularity in the ocular media may reduce the contrast sensitivity and contribute to disability glare^15^. Our study confirmed that removal of redundant conjunctiva stabilizes the tear film and improves the contrast sensitivity of patients with grade 2 or 3 temporal CCh.

Previous studies also shown that conjunctival recession and amniotic membrane transplantation, conjunctival resection and conjunctival cauterization all contributed to the relief of symptoms and ocular surface status of patients with CCh^12^,^13^,^20^. However, there was no study comparing their efficacy at the same time.

There were several limitations to our study. Firstly, the study included some elderly who may have meibomian gland disease, but the study did not evaluate the patients’ meibomian gland function. Besides, the study did not assess the patients with central and nasal CCh. Furthermore, the sample size was limited. Lastly, the study was lack of long-term observation. Further study could be conducted to solve the above issues.

In conclusion, patients with temporal CCh accompanied by dry eye present with anatomic structural changes to the lower fornix, thus leading to tear film instability and a shallow tear meniscus. The removal of redundant conjunctiva is conducive to reconstruction of the tear meniscus, loss of dry eye symptoms, improvement in tear film stability and corneal surface irregularity, and increased contrast sensitivity.

## Patients and Methods

### Patients

In this prospective study, 39 eyes from 39 individuals with temporal CCh and dryness who did not have nasal CCh and did not respond to topical medical therapy were recruited from the Department of Ophthalmology of Peking University Third Hospital from December 2013 through November 2014. Ethics committee approval was obtained from Peking University Third Hospital, and the study was conducted in accordance with the Declaration of Helsinki. Informed consent was obtained from all patients before their participation in the study. All patients underwent both objective and subjective optometry, and each individual was corrected to his or her best visual acuity.

Patients with grade 2 or 3 temporal CCh and complained of dryness were recruited, and the diagnosis of CCh was made in accordance with the criteria previously summarized by Meller and Tseng, and included grading the lid-parallel conjunctival folds (LIPCOF)^21^ (Table 4). Only one eye from each individual was included. If both eyes were eligible, the right eye of the patient was included.

Patients were excluded if they had a history of ocular surgery, any lid abnormalities, contact lens use, recent ocular infections, use of any medication or eye drops that may have affected the ocular surface within the past six months, diabetes, hay fever, or autoimmune diseases such as Sjögren’s syndrome or rheumatoid arthritis.

### Conjunctival excision

The same surgeon (Weiqiang Qiu) performed all conjunctiva excision. After sterile draping of the conjunctival sac with 0.9% saline followed by tobramycin injecion, 2% oxybuprocaine was administered as ocular superficial anesthesia. Each eye was opened with blepharostat, and 2% lidocaine was injected into the subconjunctiva. The redundant inferior bulbar conjunctiva that was 0.5 mm away from the corneal limbus was lifted up and excised as a crescent shape with ophthalmic microscissors. Then, cautery was used to adhere the conjunctiva to the underlying sclera and combine an incision margin along the corneal limbus. Postoperatively, dexamethasone and tobramysin eye drops were used four times daily for one week.

### Measurements

Participants’ symptoms were evaluated by a validated Chinese translation of the ocular surface disease index (OSDI) questionnaire at each visit^22^.

A Visante anterior segment OCT instrument (Carl Zeiss Meditec, Inc.) was used to collect the images of the cross-sectional lower tear meniscus area (TMA) before corneal staining to reduce interference with images. Subjects were asked to keep looking straight at a fixed target, with spontaneous blinking, during the examination. Immediately after blinking, the image demonstrating the central portion of both lower tear menisci and the corneal reflectivity was processed to obtain the TMA. The internal software was used to delineate the margin of the TMA variables; then, Image-Pro Plus 6.0 (Media Cybernetics, USA) was used to calculate the TMA. The same examiner conducted each examination three times, and the average results were recorded. All examinations were performed at constant temperature and humidity.

Corneal surface irregularity was then obtained through corneal topography (Wave Light Laser Technologie AG, Germany). Each eye was scanned twice, and the mean value of irregularity was recorded.

Corneal fluorescein staining was performed in a conventional manner^23^,^24^. The corneal fluorescein staining grade was classified as follows: 0 = no staining; 1 = less than 5 dots; 2 = between 1 and 3; and 3 = bulk or strip staining. The corneas were divided into four quadrants (superior temporal, inferior temporal, superior nasal, and inferior nasal), and each quadrant was scored separately and summed to a final score for each eye. At the same time, the same observer recorded the results of the tear film break-up time (TBUT) for each patient. The patients were asked to take a 10-minute rest break; then, Schirmer’s I test was conducted without anesthesia.

The OPTEC6500 functional vision testing system (Stereo Optical, USA) was used to measure the contrast sensitivity and glare disability at five spatial frequencies, 1.5, 3, 6, 12, and 18 cycles per degree (cpd).

All of the above examinations and the OSDI questionnaire were repeated, in order, three months after conjunctiva cauterization.

### Statistical analyses

Statistical analysis was conducted with the Statistical Package for Social Sciences software (version 23.0, SPSS, Inc., USA). Nonparametric tests were used to evaluate the dry eye parameters, TMA, and corneal surface irregularity. Paired Student’s *t*-test was used to identify the contrast sensitivity differences in the study parameters before and after treatment. All data are presented as the means ± standard deviations (M ± SD) for all patients. Statistically significant differences were defined as *p* ≤ 0.05.

## Additional Information

**How to cite this article**: Qiu, W. *et al*. Evaluation of the Effects of Conjunctivochalasis Excision on Tear Stability and Contrast Sensitivity. *Sci. Rep.* **6**, 37570; doi: 10.1038/srep37570 (2016).

**Publisher's note:** Springer Nature remains neutral with regard to jurisdictional claims in published maps and institutional affiliations.

## Figures and Tables

**Table 1:** General patient information.

General information	
No. of eyes (n)	36
Age (mean ± SD)	62.39 ± 6.12
Gender (male/female)	4:7
Grade of conjunctivochalasis (mean ± SD)	2.81 ± 0.36

**Table 2:** Summary of the mean values of clinical ocular surface parameters and optical coherence tomography-defined tear meniscus dimensions from pre-operation to 3 months post-operation.

	N	Pre-operation	3 months post-operation	*p* Value
Ocular surface disease index	36	58.11 ± 19.36	18.27 ± 11.67	<0.001
Tear film break-up time (s)	36	1.86 ± 0.81	5.53 ± 2.22	<0.001
Corneal fluorescein staining scores	36	1.05 ± 0.54	0.16 ± 0.15	<0.001
Schirmer’s I test (mm)	36	6.29 ± 2.93	7.92 ± 2.58	0.286
Corneal surface irregularity	36	0.026 ± 0.003	0.019 ± 0.011	0.032
Tear meniscus area (μm^2^)	36	641.28 ± 339.57	3,256.42 ± 4,884.98	0.019

**Table 3:** Contrast sensitivity in simulated daylight, night and glare disability before and after treatment.

Conditions	c/d	1.5 cpd	3 cpd	6 cpd	12 cpd	18 cpd
Daylight (85 cd/m^2^)	Before	39.98 ± 0.23	55.36 ± 0.31	42.93 ± 0.29	12.21 ± 0.16	5.19 ± 0.21
	After	66.05 ± 0.18	79.75 ± 0.27	75.81 ± 0.82	28.92 ± 0.25	12.37 ± 0.14
	*p* Value	0.001	0.012	0.005	0.003	0.001
Night (3 cd/m^2^)	Before	46.83 ± 0.24	42.16 ± 0.09	26.95 ± 0.64	3.77 ± 0.24	1.29 ± 0.01
	After	72.69 ± 0.79	78.72 ± 0.13	47.21 ± 0.52	14.91 ± 0.52	3.44 ± 0.46
	*p* Value	0.001	0.002	0.019	0.003	0.040
Glare disability	Before	28.20 ± 0.18	27.86 ± 0.51	15.96 ± 0.32	3.56 ± 0.18	0.45 ± 0.03
	After	59.07 ± 0.33	54.57 ± 0.41	39.14 ± 0.39	13.32 ± 0.52	2.93 ± 0.16
	*p* Value	<0.001	0.002	0.036	0.004	0.006

**Table 4:** Grading of conjunctivochalasis by lid-parallel conjunctival folds (LIPCOF).

Grade	Number of folds and relationship to the tear meniscus height
0	No persistent fold
1	Single, small fold
2	More than two folds and not higher than the torn meniscus
3	Multiple folds and higher than the torn meniscus
